# Initial Experience with Laparoscopic Ureteroplasty with a Spliced Appendiceal Mucosal Graft for Proximal Ureteral Stricture

**DOI:** 10.1016/j.euros.2025.10.012

**Published:** 2025-11-05

**Authors:** Zhaohui Chen, Shangjun Wu, Ming Xiong, Xiaoming Wang, Yuqi Wu, Ruixiang Dai, Xiqi Peng, Huiling Jiang, Song Wu, Teng Hou

**Affiliations:** a Department of Urology, South China Hospital, Shenzhen University, Shenzhen, China; b Department of Urology, Shenzhen People’s Hospital, Shenzhen, China; c Shenzhen Huazhong University of Science and Technology Research Institute, Shenzhen, China

**Keywords:** Spliced appendiceal mucosal graft, Ureteroplasty, Ureteral stricture

## Abstract

Long-segment proximal ureteral strictures present a significant surgical challenge, particularly in patients unwilling or unsuitable for an oral mucosal graft. Here we report the first series of laparoscopic ureteroplasty procedures with a spliced appendiceal mucosal graft (SAMG) for proximal ureteral stricture. The novel technique was performed successfully in all three patients without conversion to open surgery. The mean operative time was 178.3 min, mean estimated blood loss was 36.7 ml, and mean hospital stay was 5.6 d. The postoperative follow-up period was at least 12 mo, and all patients exhibited a reduction in hydronephrosis and no obstruction of the reconstructed ureteral segments. There were no major intraoperative or postoperative complications. SAMG ureteroplasty appears to be a safe and effective surgical method for repair of proximal ureteral stricture.

## 1 Case series

### 1.1 Introduction

The treatment of long-segment proximal ureteral strictures remains a surgical challenge. Oral mucosal graft (OMG) ureteroplasty has become a preferred option for such cases owing to its excellent tissue compatibility. However, its use is limited by donor site morbidity, including tongue pain, speech and mastication disturbances, and sensory deficits. In addition, the graft material may be unavailable in redo patients with prior oral surgery [1]. The colonic mucosa has been successfully used in urinary tract reconstruction; however, the graft harvesting procedure is complex and often requires endoscopic microsurgery [2,3]. Here we report the first case series of laparoscopic augmented anastomotic ureteroplasty using a spliced appendiceal mucosal graft (SAMG).

### 1.2 Study cohort

Three patients underwent SAMG ureteroplasty from January to March 2024. All procedures were performed by the same surgeon (T.H.). We reviewed patients’ medical histories, perioperative records, and follow-up results to summarize our preliminary findings. Investigations performed included computed tomography (CT) urography and antegrade and retrograde urography.

Patient 1 was a 36-yr-old female who presented with progressive left-sided flank pain. She had a history of ureteroscopic lithotripsy for upper ureteral calculi, after which she experienced persistent hydronephrosis despite placement of a double-J stent. Subsequent imaging confirmed a 5-cm-long proximal ureteral stricture.

Patient 2 was a 50-yr-old male who had a history of a right ureteral stone and underwent ureteroscopic holmium laser lithotripsy twice. Antegrade and retrograde urography showed a proximal ureteral stricture with a length of approximately 5 cm.

Patient 3 was a 20-yr-old male who underwent robot-assisted pyeloplasty 3 yr previously for a ureteropelvic junction obstruction, but developed a 3-cm recurrent stricture at the operative site.

All three patients declined OMG harvest because of concerns about donor site morbidity. Ultrasonography or CT was conducted to assess postoperative outcomes. The study was approved by the Ethics Committee of South China Hospital of Shenzhen University, and written informed consent was obtained from all patients.

### 1.3 Surgical technique

**Fig. 1:**
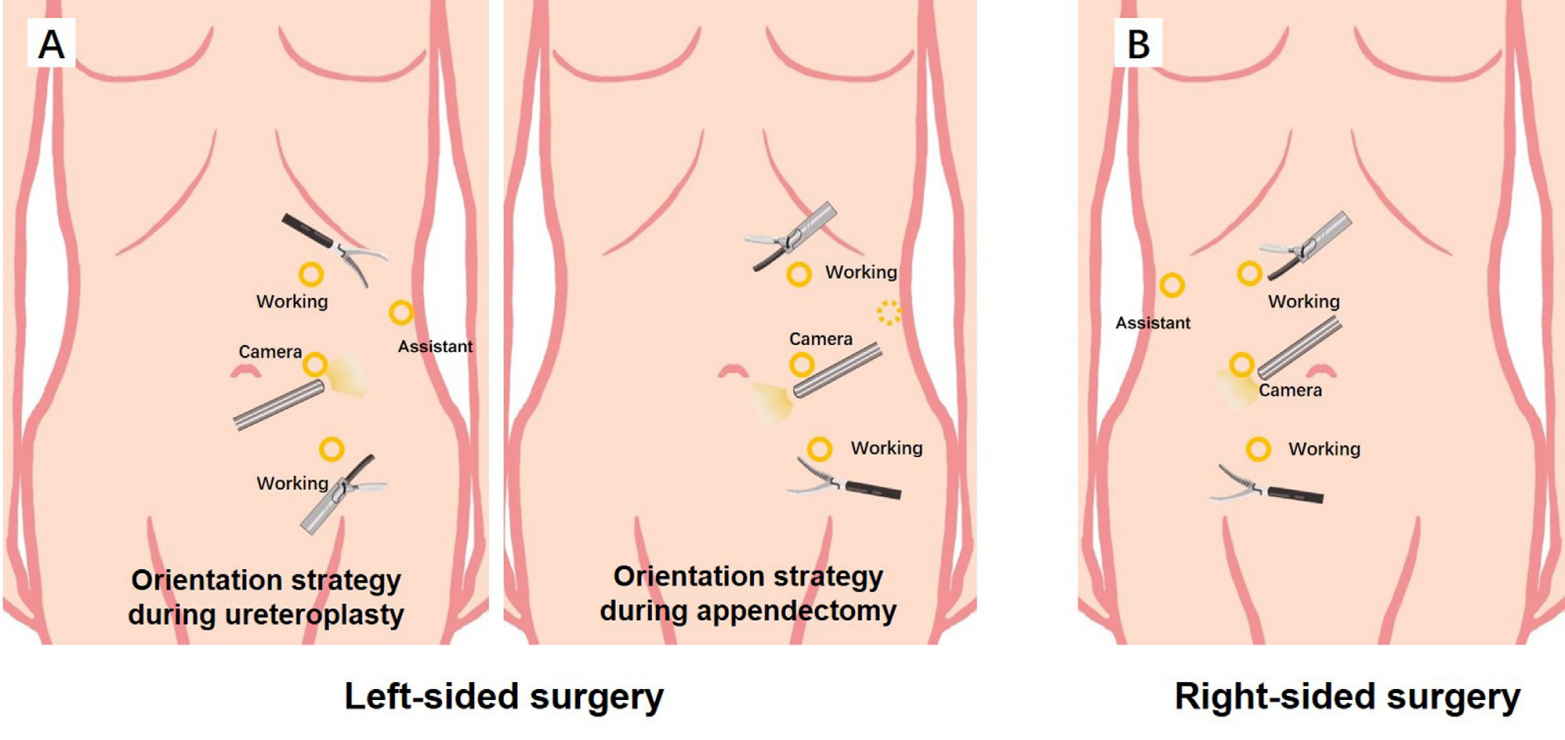
Trocar placement and orientation strategy for (A) left-sided and (B) right-sided surgery.

Left-sided surgery was performed in a modified supine position with 30° rotation of the abdomen, which facilitates a single-stage procedure without patient repositioning [4]. Right-sided surgery was performed in a 70° lateral decubitus position. A 10-mm camera trocar was inserted at the lateral margin of the rectus abdominis muscle, after which two working trocars were placed approximately 6 cm cephalic and caudal to the camera port. A 5-mm working port was placed 5 cm below the xiphoid. Another 5-mm trocar was placed 2 cm below the umbilicus when required during appendectomy (Fig. 1A,B).

**Fig. 2:**
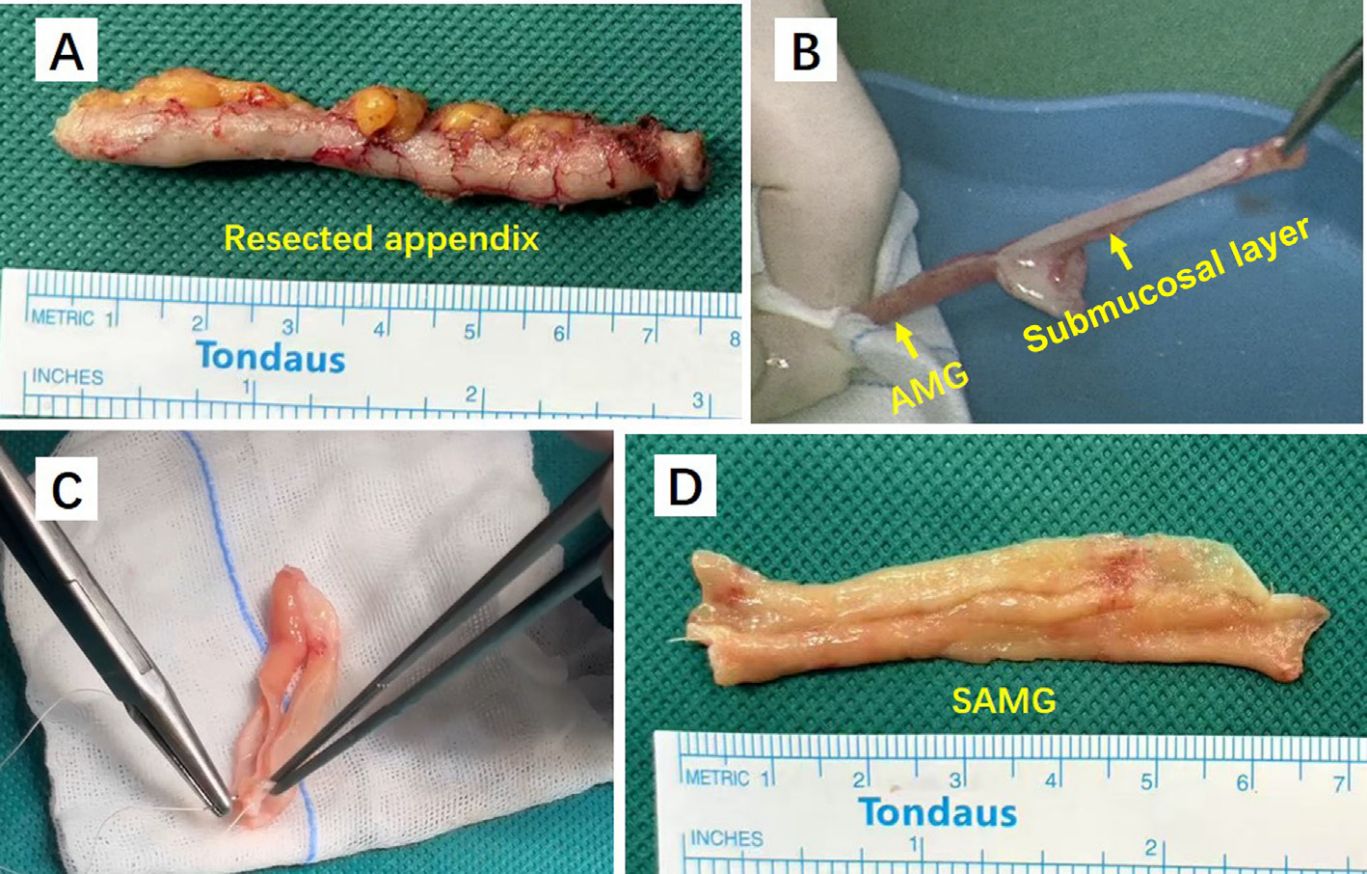
(A) Measurement of the resected appendix. (B) Isolation of the appendiceal mucosal graft (AMG) from the submucosal layer. (C) Transection of the AMG at its midpoint and reapproximation into a spliced AMG (SAMG). (D) Measurement of the SAMG.

The colon was mobilized medially and the ureter was exposed. In patient 1, we found that the stenotic ureteral segment was completely obliterated, and decided to perform augmented anastomotic ureteroplasty [5]. After resection of the obliterated segment, the proximal and distal ureters were spatulated along their ventral aspects. The kidney and distal ureter were mobilized to facilitate tension-free ureteral reconstruction. The dorsal walls of the proximal and distal ureteral stumps were anastomosed with 5-0 barbed sutures. Then the operating table was counter-rotated to achieve a neutral supine position, and the laparoscope was turned accordingly to adjust the orientation of the view for subsequent appendectomy. The appendix was identified, mobilized with its mesoappendix intact, and excised at the base using Hem-o-Lok clips. The appendix was then opened longitudinally along its antimesenteric border on the back table. Two hemostats were used to grasp the mucosal edge and the submucosal layer. With gentle counter-traction, the mucosal layer was sheared off from the submucosal plane in a controlled manner. Owing to the elasticity of the mucosa, the appendiceal mucosal graft (AMG) elongates when separated from the submucosal layer, reaching a length of 8–12 cm. The AMG is transected at its midpoint and the segments are reapproximated to form the SAMG using continuous 5-0 absorbable sutures. After suturing the two AMG pieces together, the assembled graft attains a length of 4–6 cm and a width of 1.5–2 cm (Fig. 2).

In patients 2 and 3, we made a ventral incision in the obstructed ureteral segment, with extension of the incision into the normal-width ureter for 5 mm caudally and cranially to the stricture. After determining the stricture length, we performed appendectomy and obtained an SAMG. During a right**-**sided procedure, the appendix could be accessed without requiring adjustment of the orientation of view.

**Fig. 3:**
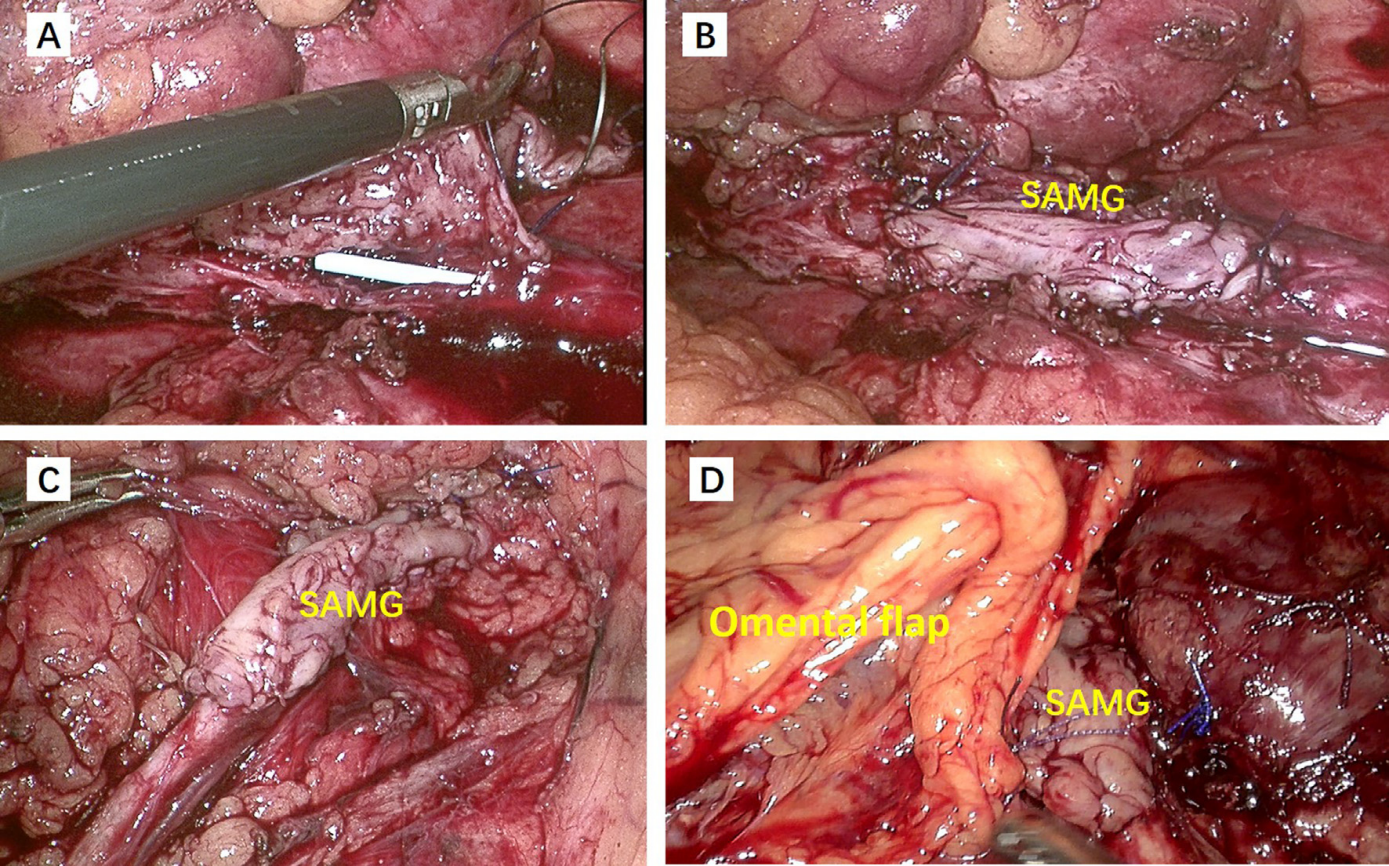
Anastomosis of the spliced appendiceal mucosal graft (SAMG) to the ventral defect of the ureter in (A,B) patient 1, (C) patient 2, and (D) patient 3. In patient 3 the reconstructed ureteral segment was wrapped with omental fat.

The SAMG was meticulously trimmed to match the measured ureteral defect length, and then anastomosed to the ventral defect using 5-0 running absorbable sutures. An antegrade 5-Fr double-J stent was inserted during the procedure. Finally, the reconstructed ureteral segment was wrapped with a vascularized omental pedicle flap (Fig. 3).

**Fig. 4:**
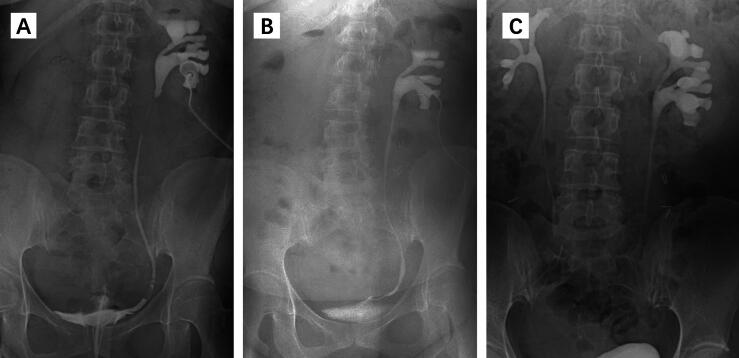
Representative perioperative and postoperative imaging. (A) Perioperative antegrade and retrograde urography showing a 5-cm-long proximal ureteral stricture. (B,C) Antegrade urography and intravenous pyelogram urography showed no obstruction in the reconstructed ureter (B) 8 wk and (C) 12 mo after surgery, respectively.

All procedures were successful. There were no intraoperative or postoperative complications. The mean operative time was 178.3 min, mean estimated blood loss was 36.7 ml, and mean hospital stay was 5.6 d (Table 1). The double-J stent was removed 8 wk after surgery. Follow-up results at a minimum of 12 mo after surgery demonstrated a reduction in hydronephrosis in all patients in comparison to their preoperative status, and the repaired ureteral segment remained unobstructed (Fig. 4).

**Table 1:** Patient information

Case	Age (yr)	Sex	Operative time (min)	EBL (ml)	SAMG length (cm)	Laterality	Surgical technique	LOS (d)
1	36	Female	218	50	5	Left	AAO	6
2	50	Male	172	40	5	Right	IO	6
3	20	Male	145	20	3	Left	IO	5

EBL = estimated blood loss; SAMG = spliced appendiceal mucosal graft; AAO = augmented anastomosis with onlay; IO = incision and onlay; LOS = length of postoperative hospital stay.

## 2 Discussion

The appendix has been used as a pedicled flap or interpositioned segment in the treatment of ureteral stricture with encouraging results but notable limitations [6,7]. Appendiceal flaps and interposition are largely restricted to right-sided ureteral strictures and may be unsuitable in patients with a short mesoappendix, which limits their applicability for proximal ureteral strictures. By contrast, the SAMG expands the clinical role of appendix-based reconstruction as the mucosal layer is used as a free graft rather than a pedicled flap. This modification allows repair of right-sided strictures, even in patients with a short mesoappendix, and, importantly, left-sided ureteral strictures, which were previously considered inappropriate for appendiceal use.

In comparison to buccal or lingual mucosal grafts, SAMG offers the advantage of avoiding oral donor-site morbidity [1]. This is particularly relevant in patients who have undergone prior oral surgery and those who are unwilling to accept oral complications. In addition, harvesting of the appendiceal mucosa is technically straightforward and performed within the same operative field, whereas colonic mucosal grafts require bowel preparation and endoscopic harvesting, which add complexity and potential risks. These features make the appendix a practical and readily accessible graft source for minimally invasive reconstructive procedures. Nonetheless, an SAMG strategy is not without drawbacks. Appendectomy carries the risk of intra-abdominal complications, particularly for surgeons unfamiliar with laparoscopic appendectomy [8]. Moreover, a short or narrow appendix may not yield an adequate graft length, which underscores the importance of preoperative evaluation.

The elasticity of the appendiceal mucosa allows significant elongation when the appendix is separated from the submucosal layer. By dividing the stretched mucosal graft at its midpoint and then splicing the segments, we reconstructed SAMGs capable of repairing 3–5-cm ureteral strictures. Furthermore, the spliced graft achieves a width of 1.5–2 cm, which ensures adequate luminal diameter in the reconstructed ureter. To the best of our knowledge, this is the first case series of laparoscopic augmented anastomotic ureteroplasty using an SAMG for a proximal ureteral stricture. The SAMG approach may extend the clinical applicability of appendiceal grafts beyond previous indications for ureteral stricture repair.

In conclusion, our preliminary findings suggest that SAMG ureteroplasty represents a novel and feasible treatment option for long-segment proximal ureteral strictures, particularly in patients unsuitable for OMG or pedicled appendiceal flap ureteroplasty. Future studies should involve larger patient cohorts to better assess the potential value and limitations of this technique.

  ***Conflicts of interest***: The authors have nothing to disclose.

  ***Funding/Support and role of the sponsor***: This study was supported by the Natural Science Foundation of Guangdong Province (no. 2023A1515011664). The funding body had no direct role in the study.
